# Metabolic engineering of *Corynebacterium glutamicum* for efficient production of optically pure (2R,3R)-2,3-butanediol

**DOI:** 10.1186/s12934-022-01875-5

**Published:** 2022-07-25

**Authors:** Mengyun Kou, Zhenzhen Cui, Jing Fu, Wei Dai, Zhiwen Wang, Tao Chen

**Affiliations:** 1 Frontier Science Center for Synthetic Biology and Key Laboratory of Systems Bioengineering of Ministry of Education, SynBio Research Platform, Collaborative Innovation Center of Chemical Science and Engineering, School of Chemical Engineering and Technology, Tianjin University, Tianjin, 300072 China; 2 Department of Biology and Biological Engineering, Chalmers University of Technology, Gothenburg, Sweden; 3 Department of Biochemical Engineering, School of Chemical Engineering and Technology, Tianjin University, Tianjin, 300072 People’s Republic of China

**Keywords:** (2R,3R)-2,3-butanediol, *Corynebacterium glutamicum*, Metabolic engineering, Microbial fermentation

## Abstract

**Background:**

2,3-butanediol is an important platform compound which has a wide range of applications, involving in medicine, chemical industry, food and other fields. Especially the optically pure (2R,3R)-2,3-butanediol can be employed as an antifreeze agent and as the precursor for producing chiral compounds. However, some (2R,3R)-2,3-butanediol overproducing strains are pathogenic such as *Enterobacter cloacae* and *Klebsiella oxytoca*.

**Results:**

In this study, a (3R)-acetoin overproducing *C. glutamicum* strain, CGS9, was engineered to produce optically pure (2R,3R)-2,3-butanediol efficiently. Firstly, the gene *bdhA* from *B. subtilis* 168 was integrated into strain CGS9 and its expression level was further enhanced by using a strong promoter *P*_*sod*_ and ribosome binding site (RBS) with high translation initiation rate, and the (2R,3R)-2,3-butanediol titer of the resulting strain was increased by 33.9%. Then the transhydrogenase gene *udhA* from *E. coli* was expressed to provide more NADH for 2,3-butanediol synthesis, which reduced the accumulation of the main byproduct acetoin by 57.2%. Next, a mutant *atpG* was integrated into strain CGK3, which increased the glucose consumption rate by 10.5% and the 2,3-butanediol productivity by 10.9% in shake-flask fermentation. Through fermentation engineering, the most promising strain CGK4 produced a titer of 144.9 g/L (2R,3R)-2,3-butanediol with a yield of 0.429 g/g glucose and a productivity of 1.10 g/L/h in fed-batch fermentation. The optical purity of the resulting (2R,3R)-2,3-butanediol surpassed 98%.

**Conclusions:**

To the best of our knowledge, this is the highest titer of optically pure (2R,3R)-2,3-butanediol achieved by GRAS strains, and the result has demonstrated that *C. glutamicum* is a competitive candidate for (2R,3R)-2,3-butanediol production.

**Supplementary Information:**

The online version contains supplementary material available at 10.1186/s12934-022-01875-5.

## Background

The increasing prices and environmental impacts of fossil fuels had made sustainably produced synthetic fuels and chemicals get more attention, which would allow us to decrease net CO_2_ emissions and realize the goal of carbon neutral. 2,3-butanediol is a high value platform chemical with a wide range of applications. It is employed as a fuel additive due to its high combustion value(27.2 kJ/g) [1]. It is also used in the manufacture of printing inks, perfumes, fumigants, moistening agents, plasticizers and antifreeze agents, etc. [2, 3]. Furthermore, 2,3-butanediol could be converted to 1,3-butadiene, methyl ethyl ketone (MEK), 2,3-butanediol diester and acetoin [2, 4–6]. And the potential global market for key downstream products of 2,3-butanediol was approximately 32 million tons per year with sales of approximately $43 billion [7].

The synthesis of 2,3-butanediol by traditional petrochemical-based processes results in gargantuan energy consumption and CO_2_ emissions [8]. Constructing microbial cell factories for the production of 2,3-butanediol from biomass is considered as a strong alternative. Extensive studies have been carried out to develop efficient microbial cell factories for producing 2,3-butanediol, including *Bacillus subtilis *[9, 10], *Bacillus amyloliquefaciens *[11, 12], *Bacillus licheniformis *[13, 14], *Klebsiella oxytoca *[15, 16], *Klebsiella pneumoniae *[17, 18], *Paenibacillus polymyxa *[19, 20], *Serratia marcescens *[21, 22], *Enterobacter cloacae *[23, 24], *Enterobacter aerogenes *[25, 26], *Escherichia coli *[3, 27], *Zymomonas mobilis *[28] and *Pichia pastoris *[29], etc. Significantly, the *K. pneumoniae* SDM isolated from orchard soil accumulated 150 g/L 2,3-butanediol at 38 h in a 5-L bioreactor, representing the highest titer achieved by the wild type [17]. However, most microorganisms with ability to naturally accumulate 2,3-butanediol produce a mixture of two of the three isomers ((2S,3S)-, (2R,3R)-, meso-2,3-butanediol) [2, 30], which limits its application. Generally the production of optically pure 2,3-butanediol can be achieved by expressing specific 2,3-butanediol dehydrogenase (BDH) in engineered strains. The highest (2R,3R)-2,3-butanediol titer to date was 152.0 g/L with an optical purity of 97.5%, which was achieved by engineered strain *E. cloacae* SDM 09 utilizing a mixture of glucose and xylose within 44 h [23]. Ge et al. [14] constructed strain *B. licheniformis* MW3 (*ΔbudC*), which accumulated 123.7 g/L (2R,3R)-2,3-butanediol with 99% optical purity at 42 h. However, some over-producing strains are conditionally pathogenic and require higher requirements for production processes which limited their potential in industrial application.

*Corynebacterium glutamicum*, a GRAS microorganism with good biotransformation properties and clear genetic backgrounds, is a very mature strain for industrial scale amino acid production [31]. Radoš et al. [32] introduced the 2,3-butanediol biosynthetic pathway of *L. lactis* into *C. glutamicum* ATCC13032 and deleted genes of competing pathway. The final strain produced 6.3 g/L 2,3-butanediol with a yield of 0.33 g/g·glucose and a productivity of 0.2 g/L/h. Yang et al. [33] constructed strain SGSC102 by metabolic engineering modification of *C. glutamicum* ATCC 13032, which could produce 18.9 g/L 2,3-butanediol from 80 g/L glucose in CGXII medium. Indeed, both the titer and yield of 2,3-butanediol achieved by *C. glutamicum* are not ideal compared with other over-producing strains. However, metabolic engineered *C. glutamicum* has exhibited excellent performance in (3R)-acetoin production with a titer up to about 100 g/L [6, 34]. Given that (3R)-acetoin is the direct precursor of 2,3-butanediol, *C. glutamicum* has great potential to produce 2,3-butanediol with high titer and yield.

**Fig. 1:**
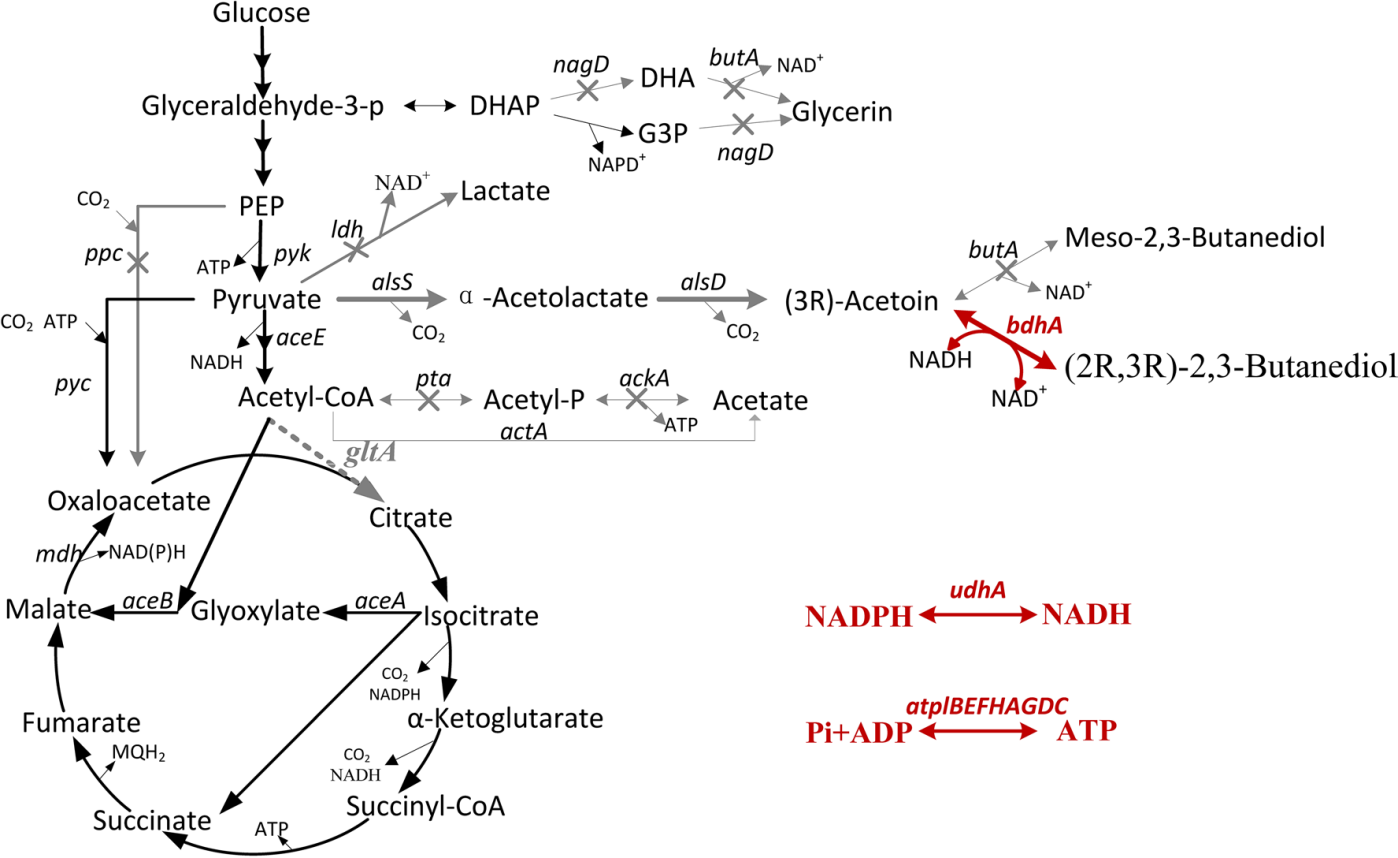
The (2R,3R)-2,3-butanediol biosynthesis pathway of *C. glutamicum*. Genes manipulated in this study are indicated in red. The bold arrows indicate metabolic fluxes increased by overexpression of the corresponding genes. The gray arrows indicate the reactions leading to a byproduct or presumably irrelevant reactions. Deleted genes are indicated with crosses. Downregulated genes are indicated with dashed arrows. GAP: glyceraldehyde-3-phosphate; DHAP: dihydroxyacetone phosphate; DHA: dihydroxyacetone; G3P: sn-glycerol 3-phosphate; PEP: phosphoenolpyruvate; OAA: oxaloacetate. Genes and their encoded enzymes: *alsS*, acetolactate synthase; *alsD*, acetolactate decarboxylase; *ppc*, phosphoenolpyruvate carboxylase; *pyc*, pyruvate carboxylase; *icd*, isocitrate dehydrogenase; *gltA*, citrate synthase. *pta*, phosphotransacetylase; *ackA*, acetate kinase; *aceE*, E1 component of the pyruvate dehydrogenase complex; *nagD*, putative phosphatase; *butA*, meso-2,3-butanediol dehydrogenase; *bdhA*, (2R,3R)-2,3-butanediol dehydrogenase; *udhA*, transhydrogenase; *atplBEFHAGDC*, *atp* operon structure, *atpI*, hypothetical protein; *atpB*, a subunit of H^+^-ATPase synthase; *atpE*, c subunit; *atpF*, b subunit; *atpH*, δ subunit; *atpA*, α subunit; *atpG*, γ subunit; *atpD*, β subunit; *atpC*, ε subunit

In this study, *C. glutamicum* CGS9, a (3R)-acetoin over-producing strain, was used to be further engineered for (2R,3R)-2,3-butanediol production. It can produce15.70 g/L (3R)-acetoin in flask culture with a yield of 0.408 g/g glucose, which was 83.4% of the theoretical yield [6]. A series of metabolic engineering strategies were adopted to enhance the (2R,3R)-2,3-butanediol production (Fig. 1), and the final engineered strain CGK4 produced 144.9 g/L 2,3-butanediol with a yield of 0.429 g/g glucose and a productivity of 1.10 g/L/h in fed-batch fermentation. Significantly, the optical purity of the resulting (2R,3R)-2,3-butanediol surpassed 98%. To the best of our knowledge, this is the highest level of production of (2R,3R)-2,3-butanediol using GRAS strains to date, making it a competitive (2R,3R)-2,3-butanediol producer.

## Results and discussion

### Construction of (2R,3R)-2,3-butanediol generation pathway and optimization of the expression level of butanediol dehydrogenases

CGS9 [6] is a (3R)-acetoin overproducing strain with three copies of the *alsSD* operon in the genome, in which biosynthesis pathways of the major by-products were disrupted and the TCA cycle was weakened by downregulating the expression of the *gltA* gene. In order to convert (3R)-acetoin to (2R,3R)-2,3-butanediol, the gene *bdhA* encoding 2,3-butanediol dehydrogenase from *B. subtilis* 168, under control of the constitutive promoter *Ptrc* (without *lacO* sequence), was inserted into the genome of CGS9 at the *∆ldh* locus, generating the strain CGK1. Strains were cultivated in CGXIIP medium containing 40 g/L glucose. As shown in Fig. 3A, the final (2R,3R)-2,3-butanediol production by strain CGK1 reached 12.38 g/L, with a yield of 0.341 g/g glucose at 24 h. As expected, the by-products acetate (0.16 g/L), lactic acid (0.35 g/L), glycerin (0.14 g/L) and succinate (< 0.01 g/L) were all at low concentrations at 24 h, benefiting from the deletion of relevant genes [6]. It was noticed that (3R)-acetoin was the main by-product and its titer reached 3.50 g/L at 24 h, which may be caused by higher gene dosage of *alsSD* (3 copies) and the relative lower gene dosage of *bdhA* (1 copy). Consequently, the enzyme activity of BDH could be not sufficient for converting all the (3R)-acetoin into (2R,3R)-2,3-butanediol. It was found that (2R,3R)-2,3-butanediol was converted to (3R)-acetoin when glucose is depleted, which due to that the NADH produced from glycolysis was not enough and 2,3-butanediol can be converted to acetoin to regenerate NADH to maintain a constant oxidation-reduction state [35].

**Fig. 2:**
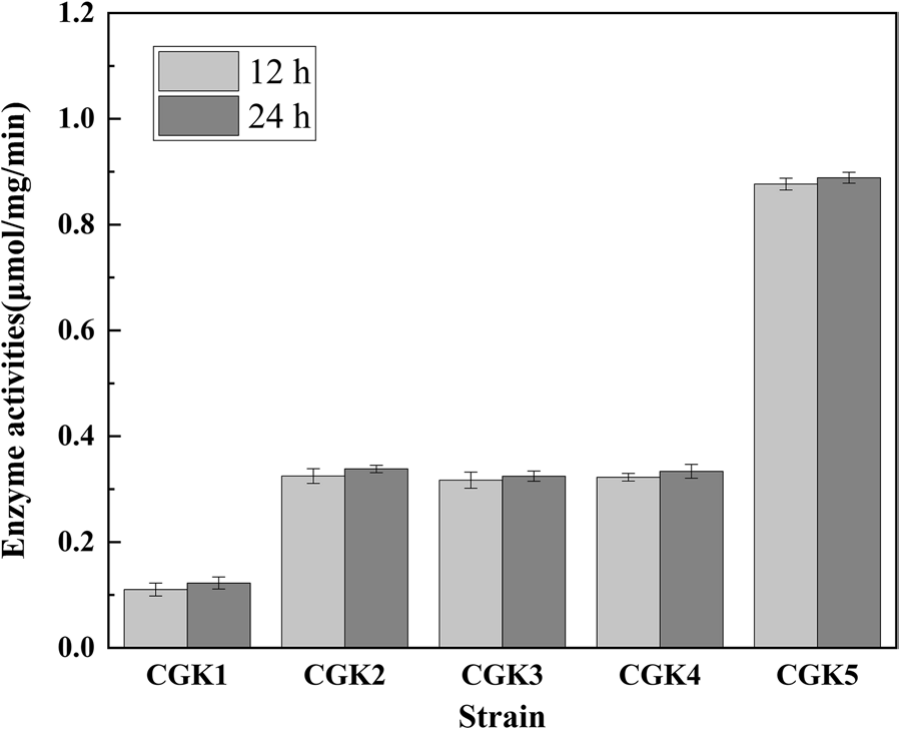
The activity of 2,3-butanediol dehydrogenase in CGK1, CGK2, CGK3, CGK4 and CGK5 at 12 and 24 h

**Fig. 3:**
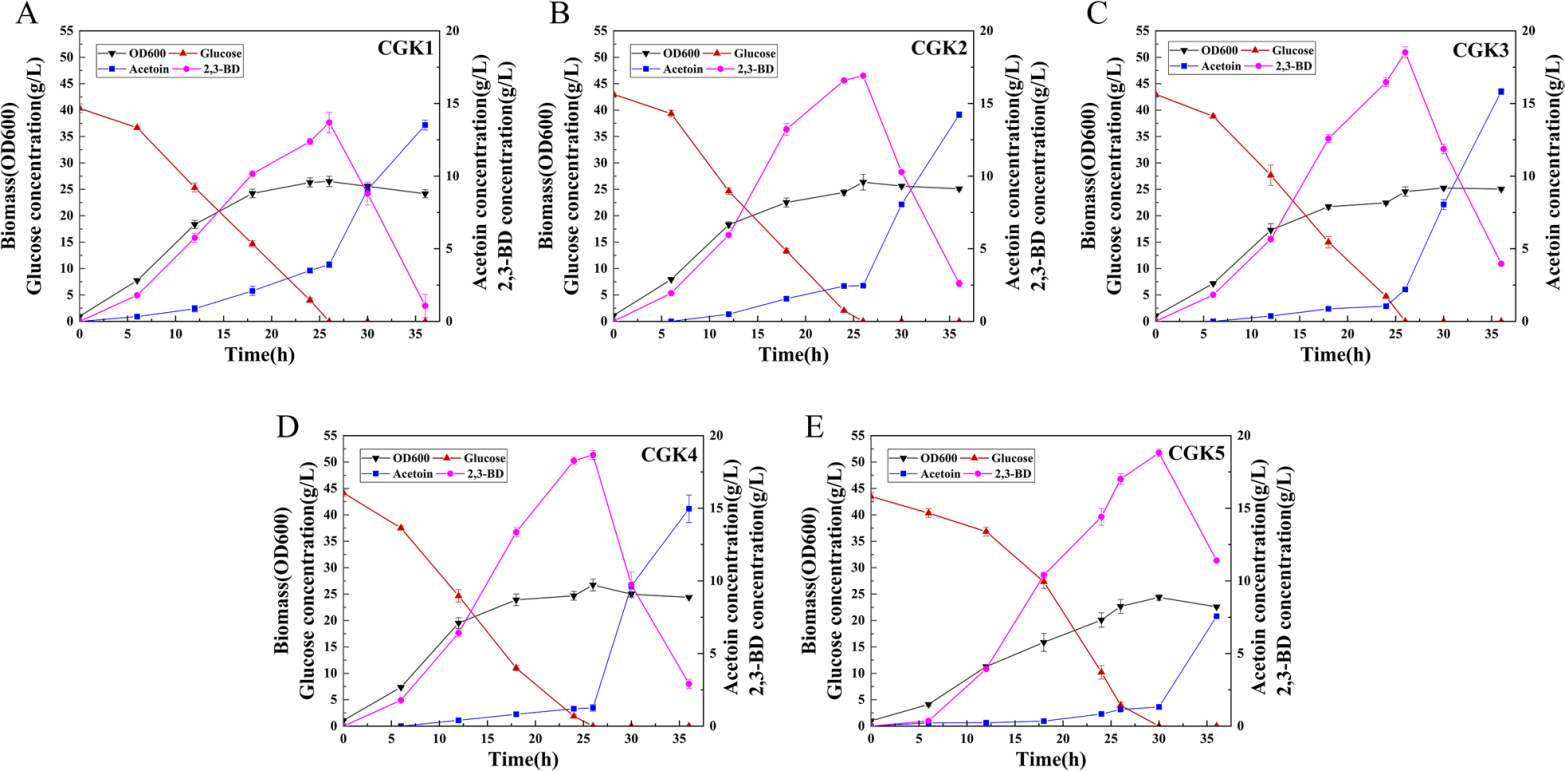
Time profiles of the biomass (OD_600_), glucose, (3R)-acetoin and (2R,3R)-2,3-butanediol (2,3-BD) concentrations of strains CGK1 (**A**), CGK2 (**B**), CGK3 (**C**), CGK4 (**D**) and CGK5 (**E**) cultured in CGXIIP medium

The expression level of heterologous genes was affected by several factors, including gene dosage, promoter strength, secondary structure of mRNA and RBS sequence [36]. To improve the expression level of *bdhA*, its promoter *Ptrc* and RBS in genome of CGK1 were replaced by the strong promoter *Psod* and RBS-10, which was designed for *bdhA* with the highest translation initiation rate (TIR). The resulting strain CGK2 showed a significant increase in BDH enzyme activity and (2R,3R)-2,3-butanediol production. As shown in Fig. 2. the BDH activity of CGK2 was 1.95-fold higher than that of CGK1 at 12 h (exponential phase) and still remained at a high level at 24 h (stable phase). Strain CGK2 produced 16.58 g/L (2R,3R)-2,3-butanediol with a yield of 0.405 g/g glucose, which was 18.8% higher than that of CGK1. Meanwhile, (3R)-acetoin titer was decreased to 2.43 g/L (Fig. 3B) but was still at a higher level, most likely due to the insufficient of reducing power in the form of NADH. The total yield of (2R,3R)-2,3-butanediol and (3R)-acetoin of CGK2 reached 0.455 g/g glucose at 24 h, which was 5.8% higher than that of CGK1 (*P* < 0.05, *t*-test). These results suggested that carbon flux was further directed toward (2R,3R)-2,3-butanediol synthesis by improving the expression level of *bdhA*.

### Improvement of NADH supply for reducing the accumulation of (3R)-acetoin byproduct

The conversion between (2R,3R)-2,3-butanediol and (3R)-acetoin could regulate the redox balance [34]. The *udhA* gene from *E. coli* W1485 encodes a transhydrogenase that partially converts NADPH to NADH, and the latter is beneficial to the conversion of (3R)-acetoin to (2R,3R)-2,3-butanediol [10]. The artificial operon driven by the strong promoter *P*_*sod*_, consisting of the genes *bdhA* and *udhA* with RBS-10, was introduced into the chromosome of CGS9 at the *∆ldh* locus to generate strain CGK3.

**Fig. 4:**
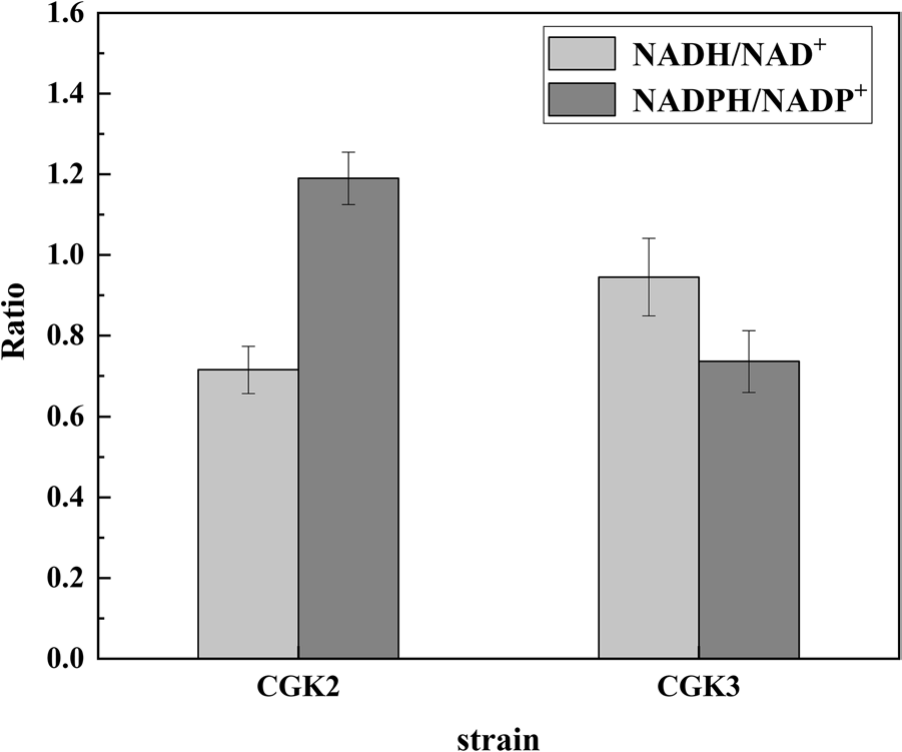
Changes in NADH/NAD^+^ and NADPH/NADP^+^ ratio in CGK2 and CGK3 at 12 h

As expected, comparing the reducing power in strains CGK2 and CGK3 at 12 h, the NADPH/NADP^*+*^ ratio significantly reduced from 1.19 to 0.74, and the NADH/NAD^*+*^ ratio increased from 0.72 to 0.94 (Fig. 4), which demonstrated the ability of *udhA* to regulate NADPH/NADP^*+*^ ratio and provided a strategy for the regulation of cofactor. As shown in Fig. 3C, strain CGK3 accumulated 16.47 g/L (2R,3R)-2,3-butanediol and its yield increased by 6.2%, reaching 0.430 g/g glucose at 24 h. The (3R)-acetoin titer of CGK3 was 1.04 g/L at 24 h, which decreased by 57.2% compared with that of CGK2. In addition, there was no significant difference in the activities of BDH between CGK2 and CGK3 (Fig. 2). It was founded that the total yield of (2R,3R)-2,3-butanediol and (3R)-acetoin decreased slightly. Meanwhile, the glucose consumption rate of CGK3 decreased by 6.6% compared with that of CGK2 (Table 1, *P* < 0.05, *t*-test), which might due to that the growth of CGK3 was inhibited by the change of reducing power. Thus, the glucose consumption rate needs to be improved, which can lead to increase in (2R,3R)-2,3-butanediol productivity.

**Table 1:** Fermentation characteristics of *C. glutamicum* strains cultivated in CGXIIP medium supplemented with initial 40 g/L glucose measured at 24 h

Strain	Biomass (OD600)	Consumed glucose (g/L)	Acetoin (g/L)	2,3-Butanediol (g/L)	2,3-Butanediol Yield (g/g glucose)	Acetoin + 2,3-Butanediol Yield (g/g glucose)
CGK1	26.30 ± 0.54	36.30 ± 0.45	3.50 ± 0.18	12.38 ± 0.21	0.341 ± 0.008	0.430 ± 0.005
CGK2	24.42 ± 0.52	40.95 ± 0.09	2.43 ± 0.02	16.58 ± 0.13	0.405 ± 0.004	0.455 ± 0.010
CGK3	22.45 ± 0.25	38.26 ± 0.28	1.04 ± 0.03	16.47 ± 0.31	0.430 ± 0.010	0.448 ± 0.008
CGK4	24.68 ± 0.83	42.28 ± 0.31	1.20 ± 0.03	18.27 ± 0.26	0.432 ± 0.010	0.451 ± 0.012
CGK5	20.08 ± 1.35	33.30 ± 1.26	0.84 ± 0.04	14.40 ± 0.57	0.432 ± 0.011	0.448 ± 0.013

Acetoin + 2,3-Butanediol yield was calculated by converting 2,3-butanediol to acetoin and adding the content of acetoin

Data are average values and standard deviations of triplicate experiments

### Decrease in ATP content to increase glucose consumption rate

It was observed that microorganisms could increase the glycolytic flux to compensate for the lack of ATP, which improved the consumption of glucose [37]. Therefore, a strategy for reducing the biosynthesis of ATP was taken to increase the glucose consumption rate of strain CGK3. It was generally considered that the bacterial *atp* operon structure consisted of the gene *atpI*, *atpB*, *atpE*, *atpF*, *atpH*, *atpA*, *atpG*, *atpD* and *atpC *[38], and the γ subunit of H^+^-ATPase encoded by the gene *atpG* worked as the main shaft for the rotation of the H^+^-ATPase rotor [39]. According to a previous report, the activity of H^+^-ATPase was reduced to 70% of the original by replacing T to C at 817 bp and C to T at 818 bp of *atpG* in *C. glutamicum* ATCC14067, which resulted in an increase of 24% in specific glucose consumption during the exponential phase [38]. Thus, the mutations were introduced into strain CGK3 to test the effect on (2R,3R)-2,3-butanediol production, yielding strain CGK4.

As shown in Fig. 3D, the glucose consumption of per cell of strain CGK4 increased by 11.8% and reached 1.05 g/L/OD during the exponential phase (0-12 h), which was lower than that described in the report [38]. It might result from the different original strain ATCC13032, instead of ATCC14067, used in this study. Another reason might be the different genotype of CGK3, in which several pathways for byproducts synthesis were blocked and the TCA cycle was weakened. The whole glucose consumption rate of CGK4 was 10.5% higher than that of CGK3 (42.28 vs. 38.26 g/L, Table 1). Correspondingly, the (2R,3R)-2,3-butanediol titer of CGK4 reached 18.27 g/L with an increase of about 10.9% in productivity at 24 h, and the yield of (2R,3R)-2,3-butanediol was almost the same as that of CGK3 (0.432 g/g glucose), which was about 86% of the theoretical yield. The titer of acetoin was 1.2 g/L, which was still at a low level.

### Effect on (2R,3R)-2,3-butanediol production by overexpressing *bdhA* and *udhA*

To test if the increase of the expression level of *bdhA* and *udhA* can further enhance the production and yield of (2R,3R)-2,3-butanediol, an episomal plamid pECK1 with additional copy of *bdhA-udhA* was constructed and introduced into strain CGK4, generating strain CGK5. As shown in Fig. 3E, the growth and glucose consumption rate of CGK5 were obviously decreased compared with that of CGK4, and decreased (2R,3R)-2,3-butanediol production was obtained with a titer of 14.41 g/L at 24 h. When glucose was depleted at 30 h, the final (2R,3R)-2,3-butanediol titer reached 18.81 g/L. Although strain CGK5 showed a litter higher (2R,3R)-2,3-butanediol titer than CGK4 (18.65 g/L at 26 h, Fig. 3D), its productivity decreased by 12.7% compared with strain CGK4. It was presumed that the metabolic burden exerted by the expression plasmid and the imbalance of NADH resulted in the decrease of growth and glucose consumption rate (Table 1). The BDH activities of CGK5 were 1.72-fold and 1.66-fold higher than those of CGK4 at 12 and 24 h, respectively, but the final yield of (2R,3R)-2,3-butanediol was similar when the glucose was depleted, which indicated the expression level of *bdhA* and *udhA* in CGK4 was sufficient to convert (3R)-acetoin to (2R,3R)-2,3-butanediol. Considering the stability of the production strain and bio-safety, strain CGK4 without plasmid and marker gene (antibiotic resistance gene) was chosen to further produce (2R,3R)-2,3-butanediol in fed-batch fermentation.

### Fed‑batch fermentation of CGK4 to produce (2R,3R)-2,3-butanediol

**Fig. 5:**
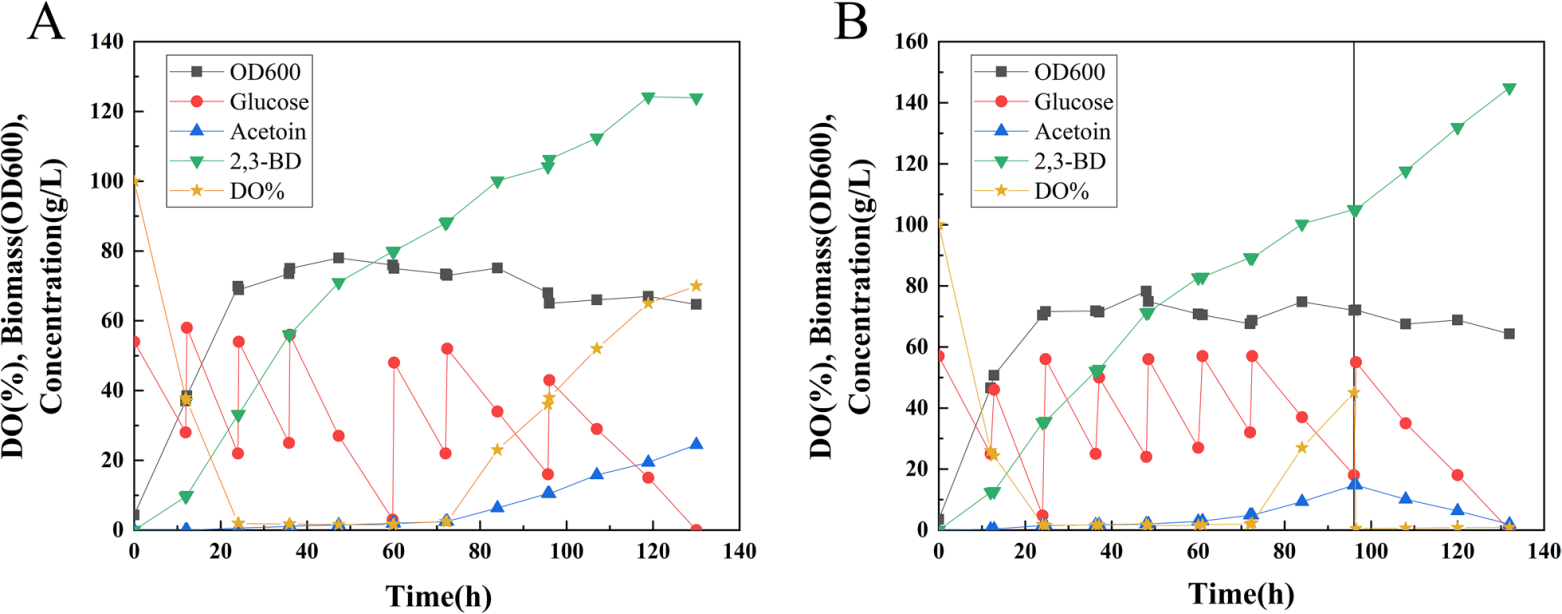
**A **Strain CGK4 was cultured in LBRC medium in fed-batch fermentation at 30 °C and 350 rpm in a 5-L fermenter under aeration of 1 vvm. **B** When the titer of acetoin reached 15 g/L, the aeration and rotational speed were adjusted to keep the dissolved oxygen between 0.5–1%. A stock solution comprising 1000 g/L glucose was added when the glucose concentration dropped below 20 g/L to keep the glucose concentration between 20 and 60 g/L

To evaluate potential of CGK4 for further industrial application, strain CGK4 was cultured in LBRC medium [34] in a 5-L fermenter without the addition of antibiotics for fed-batch fermentation. As shown in Fig. 5A, a titer of 124.2 g/L (2R,3R)-2,3-butanediol was obtained at 119 h with a productivity of 1.04 g/L/h. It was found that (2R,3R)-2,3-butanediol concentration no longer increased after 119 h, and there was still 15 g/L of glucose in the fermenter. The (2R,3R)-2,3-butanediol yield was 0.410 g/g glucose, which was 82% of the theoretical yield. During the first 24 h of fermentation, the biomass grew rapidly to an OD_600_ of 70, and dissolved oxygen also dropped to less than 2%, and then fluctuated between 1 and 2%. The dissolved oxygen gradually increased to 70% from 72 to 130 h. Meanwhile, the (3R)-acetoin production increased significantly during this stage and reached a final concentration of 24.50 g/L at 130 h. It can be inferred that the increased dissolved oxygen during 72–130 h resulted in an increase in NADH consumption by respiration, and a shortage in NADH supply for the conversion of acetoin to 2,3-butanediol.

It is well known that control of oxygen supply is the most critical factor for efficient production of 2,3-butanediol [40]. The anaerobic conversion of glucose to 2,3-butanediol is not feasible due to redox imbalance (one NADH is produced in excess) [41]. Therefore, the oxygen supply was adjusted to maintain a microaerobic condition during the fermentation to reduce the NADH consumption by respiratory. When the titer of (3R)-acetoin reached about 15 g/L (about 96 h), the aeration and rotational speed were adjusted to keep the dissolved oxygen between 0.5 and 1%. As shown in Fig. 5B, a final titer of 144.9 g/L (2R,3R)-2,3-butanediol with a yield of 0.429 g/g glucose and a productivity of 1.10 g/L/h was obtained by strain CGK4, and the optical purity of (2R,3R)-2,3-butanediol was over 98% (Additional file 1: Fig. S1). In addition, the final concentrations of the by-products α-ketoglutarate, glycerin, acetate and acetoin were 1.61, 1.49, 1.75 and 1.93 g/L, respectively. No succinate and lactate were detected. To the best of our knowledge, this is the highest titer of (2R,3R)-2,3-butanediol production achieved by GRAS strains.

However, there is still much room to improve the (2R,3R)-2,3-butanediol productivity of *C. glutamicum* compared with other over-producing strains (Table 2). It was reported that productivity of the target product is closely related to the carbohydrate uptake capacity [42]. In our future study, further improvement may be achieved by constructing an ATP futile cycle system [43] or enhancing the phosphoenolpyruvate-carbohydrate phosphotransferase system (PTS), which is the major carbohydrates uptake system in *C. glutamicum *[44].

**Table 2:** Summary of literature on microbial fermentation for the optically pure (2R,3R)-2,3-butanediol production above 100 g/L

Strains	Substrates	Titer (g/L)	Purity (%)	Yield (g/g substrates)	Productivity (g/L/h)	Refs.
*Corynebacterium glutamicum* CGK4	Glucose	144.9	98	0.43	1.10	This study
*Enterobacter cloacae* SDM 09	Glucose and xylose	152.0	97.5	0.49	3.5	[23]
	Corn stover hydrolysate	119.4	96	0.48	2.3	
*Saccharomyces cerevisiae* JL0432	Glucose and galactose	100	98	0.35	0.33	[45]
*Bacillus licheniformis* MW3*△budC*	Glucose	123.7	99	NM	2.95	[14]
*Escherichia coli* MQ1	Glucose	115	99	0.42	1.44	[46]
*Paenibacillus polymyxa* DSM 365	Sucrose	111	98	0.48	2.06	[20]
*Klebsiella oxytoca* *ΔldhAΔpflB* *ΔbudC*::PBDH(pBBR-PBDH)	Glucose	106.7	92	0.40	3.1	[47]
*Bacillus subtillis* FJ-4	Glucose	100.0	99.9	0.44	0.6	[48]

## Conclusions

In summary, a series of promising (2R,3R)-2,3-butanediol producers were constructed by metabolic engineering of a (3R)-acetoin overproducing strain. The best engineered strain CGK4 produced 144.9 g/L (2R,3R)-2,3-butanediol with a yield of 0.429 g/g glucose at a rate of 1.10 g/L/h in a 5-L fermenter and the optical purity was over 98%, which is the highest level of (2R,3R)-2,3-butanediol production in GRAS strains at present. This study provided the possibility of microbial economically viable production of (2R,3R)-2,3-butanediol by GRAS microorganism at industrial level.

## Materials and methods

### Reagents, strains and media

Primers were synthesized by GENEWIZ (Suzhou, China). Plasmids were extracted using the Axyprep™ Plasmid Miniprep Kit (Axygen, USA) and DNA was perfied using the SanPrep Column Plasmid Mini-Prep Kit (Sangon Biotech, Shanghai, China). BHI broth was purchased from Hopebio (Qingdao, China). Yeast extract was purchased from Angel (Hubei, China). 2,3-butanediol and acetoin standards were purchased from Sigma (Merck, USA). Other reagents were purchased from Sangon Biotech (Shanghai, China).

The original strain was CGS9 (*C. glutamicum* ATCC 13,032 *∆pta∆ack∆ldh∆buta∆nagD∆ppc*, *∆ackA::P*_*tuf*_ -*alsSD*, *∆butA::P*_*tuf*_*-alsSD*, *∆nagD::P*_*tuf*_*-alsSD*, *P*_*1*_-*gltA*) [6]. Strains and plasmids used in this study are listed in Table 3. *Escherichia coli* DH5α was used for plasmid construction and was grown in LB medium. BHI broth (74 g/L) was used for the tube culture and transformation of *C. glutamicum*. CGIII medium was used for pre-cultures. Batch fermentation of 2,3-butanediol was conducted in CGXIIP medium [6]. LBRC medium [6] with the indicated amounts of glucose was used in fed-batch fermentation, and the feeding medium comprising 1000 g/L glucose was prepared according to previous report [34]. Antibiotics were added where appropriate as follows: for *C. glutamicum*, kanamycin 25 mg/L, for *E. coli*, kanamycin 40 mg/L.

**Table 3:** Strains and plasmids used in this study

	Relevant characteristics	References
*Strain/plasmid*		
*E. coli* DH5α	Host for plasmid construction	Lab stock
*B. subtilis* 168	The *bdhA* gene donor	Lab stock
*E. coli* W1485	The *udhA* gene donor	Lab stock
CGS9	ATCC13032*∆pta∆ack∆ldh∆buta∆nagD∆ppc*, *∆ackA::P*_*tuf*_ -*alsSD*, *∆butA::P*_*tuf*_ -*alsSD*, *∆nagD::P*_*tuf*_ -*alsSD, P*_*1*_-*gltA*	[6]
CGK1	CGS9 *∆ldh::Ptrc*-*bdhA*	This study
CGK2	CGS9 *∆ldh::Psod*-*bdhA*	This study
CGK3	CGS9 *∆ldh::Psod* -*bdhA*-*udhA*	This study
CGK4	CGK3 *atpG*^*T817C*, *C818T*^	This study
CGK5	CGK4 pECK1	This study
*Plasmids*		
pD-sacB	Kan^R^; vector for in-frame deletion (*sacB*_*B.sub*_.; *lacZα*; *OriV*_*E.c*_.)	Lab stock
pD-sacB-*ldh*	Kan^R^; pD-sacB carrying the flanking sequences of the *ldh* gene	[34]
pD-*ldh*-A1	Kan^R^ containing *P*_*trc*_-*bdhA* flanks	This study
pD-*ldh*-A2	Kan^R^ containing *P*_*sod*_-*bdhA* flanks	This study
pD-*ldh*-AU	Kan^R^ containing *P*_*sod*_-*bdhA*-*udhA* flanks	This study
pD-sacB-*atpG*^*T817C, C818T*^	KanR, containing the sequence for *atpG* exchange T817C, C818T	This study
pEC-XK99E	Kan^R^; *C. glutamicum*/*E. coli* shuttle vector (*P*_*trc*_, *lacIq*; pGA1, *OriV*_*C.g.*_, *OriV*_*E.c.*_)	Lab stock
pECK1	derived from pEC-XK99E with *lacIq* deleted, for the overexpression of *bdhA* and *udhA* under the control of the promoter *P*_*sod*_ and *rbs designed for bdhA*	This study

### Construction of plasmids and strains

All the primers used in this study are listed in Additional file 1: Table S1. All DNA manipulations, including restriction enzyme digestion and vector isolation were carried out using standard protocols [49]. The suicide plasmid pD-sacB was used for genome editing in *C. glutamicum* via two-step homologous recombination [50].

To integrate the *P*_*trc*_-*bdhA* into the chromosome, the plasmid pD-*ldh-*A1 was constructed as follows: promoter *P*_*trc*_ was amplified from the plasmid pEC-XK99E using the primer pair *trc*-F/R, and *bdhA*1-F/R was used to amplify the *bdhA* gene from *B. subtilis* 168, after which these fragments were fused using *trc*-F/*bdhA*1-R. The resulting fragment was digested with *Sda*I/*Sal*I and ligated between the corresponding sites of pD-sacB-*ldh*. AAAGGAGGACAACC was used as RBS sequence in plasmid pD-*ldh-*A1, which was used in our previous studies [51]. The plasmids pD-*ldh-*A2 and pD-*ldh-*AU were constructed analogously to integrate the *P*_*sod*_-*bdhA* and *P*_*sod*_-*bdhA-udhA* into the chromosome respectively. The RBS sequence of these two plasmids was RBS-10 (ACGAGAAAAAAATTCGAACCCGAGAAAGGAGGTATT) designed by using the RBS Calculator (design mode) of De Novo DNA for the *bdhA* with the highest target translation initiation rate (TIR).

To introduce mutations into the endogenous gene *atpG*, the plasmid pD-sacB-*atpG*^*T817C, C818T*^ was constructed as follows: the flanking regions of the *atpG* gene with relevant modifications were amplified from genomic DNA of *C. glutamicum* using the primer pairs *atpG*U-F/R and *atpG*D-F/R. The corresponding flanking fragments were fused using *atpG*U-F/*atpG*D-R. The fused product was digested with XbaI*/*SphI and ligated between the corresponding sites of pD-sacB to construct pD-sacB-*atpG*^*T817C*, *C818T*^.

To construct the plasmid pECK1, the primer pair AU1-F/R was used to amplify the artificial operon *P*_*sod*_*-bdhA-udhA* from the plasmid pD-*ldh-*AU and pec-F/R was used to amplify the pEC-XK99E, then the plasmid pECK1 was constructed by the method of CPEC.

### Fermentation conditions

Single colonies were used to inoculate 5 mL BHI medium and grown at 220 rpm overnight to prepare *C. glutamicum* pre-cultures, and then 1 mL of the resulting seed culture was transferred into 50 mL CGIII medium with 20 g/L glucose and grown at 220 rpm for 12 h. For batch fermentation, the seed culture was used to inoculate a 250-mL shake flask containing 50 mL CGXIIP medium to an initial OD_600_ of 1 and grown at 180 rpm on a rotary shaker. All fermentations were performed at 30 °C.

For fed-batch fermentation, 200 mL of CGIII seed culture was used to inoculate a 5-L fermenter (Bailun, Shanghai, China) containing 1.8 L LBRC medium. The agitation speed was maintained at 350 rpm. All cultivations were carried out at 30 °C with an aeration rate of 1 vvm. The initial pH of the medium was 7.0. During the fermentation process, the pH value was not controlled. Fed-batch fermentation was conducted by an interim feeding method. The initial glucose concentration was 50 g/L, and an appropriate amount of feeding medium was added to maintain its concentration between 20 and 60 g/L.

### Analytical methods

Cell growth was determined by measuring the optical density at 600 nm (OD_600_) using a UV–Vis spectrophotometer. Glucose was measured using an SBA Bio-analyzer (Shandong Academy of Sciences, China). Metabolite concentrations were determined by HPLC using an HPX-87 H (300 mm × 7.8 mm) organic acid, as described previously [6]. The optical purity of 2,3-butanediol was determined by GC as described previously [9]. Samples were extracted with the same volume of ethyl acetate, and then analyzed by GC-FID (PERSEE, Beijing, China) equipped with an HP-chiral 20b column (30 m, 0.32 mm internal diameter, 0.25-mmfilm thickness). The oven temperature program was as follows: 40 °C (2 min), increased to 75 °C (4 min) at 5 °C min^− 1^, followed by a ramp of 1 °C min^− 1^ to 80 °C (2 min), and finally 15 °C min^− 1^ to 230 °C (4 min). Nitrogen was used as the carrier gas. The temperature of injector and detector were set at 230 °C.

### Determination of NADH, NAD^+^, NADPH and NADP^+^

NADH, NAD^+^, NADPH and NADP^+^ were extracted from the cells for measurement when the engineered strain grown to the exponential stage (12 h). The intracellular NADH and NAD^+^ contents were determined using the Coenzyme I NAD(H) Content Assay Kit (Solarbio, Beijing, China), and the intracellular NADPH and NADP^+^ contents were determined using the Coenzyme II NADP(H) Content Assay Kit (Solarbio, Beijing, China).

### Enzyme activity assays

The crude enzyme solution was extracted when the growth of the engineered strain to the exponential growth phase, and the method was as described previously [6]. Total protein concentrations were determined according to the Bradford method [52]. The 2,3-butanediol dehydrogenase activity was assayed by measuring the consumption of NADH as described previously [53].

## Supplementary Information

**Additional file 1: Figure S1.** Identification of2,3-butanediol enantiomers by GC-FID. A: The optically pure standards of(2S,3S)-2,3-butanediol, (2R,3R)-2,3-butanediol and meso-2,3-butanediol hadretention times of 22.957, 23.098 and 23.973 min; B: Fermentation products ofCGK1 in CGXIIP medium; C: Fed-batch fermentation products of CGK4 in LBRCmedium at 132 h; D: The standard of 2,3-butanediol ((2S,3S)-2,3-butanediol:(2R,3R)-2,3-butanediol: meso-2,3-butanediol= 0: 98: 2). E: Fermentationproducts of CGK4 in CGXIIP medium. 1: (2S,3S)-2,3-butanediol, 2:(2R,3R)-2,3-butanediol, 3: meso-2,3-butanediol. **Table S1.** Primers used in this study.

## Data Availability

All data generated or analysed during this study are included in this published article and its Additional file.
