# Supplementary material for: Metabolic engineering of Corynebacterium glutamicum for efficient production of optically pure (2R,3R)-2,3-butanediol
Source: Microb Cell Fact. 2022 Jul 25;21:150. doi: 10.1186/s12934-022-01875-5 (PMC9310479; doi:10.1186/s12934-022-01875-5)
Supplement: Supplementary file 1 — Additional file 1: Figure S1. Identification of2,3-butanediol enantiomers by GC-FID. A: The optically pure standards of(2S,3S)-2,3-butanediol, (2R,3R)-2,3-butanediol and meso-2,3-butanediol hadretention times of 22.957, 23.098 and 23.973 min; B: Fermentation products ofCGK1 in CGXIIP medium; C: Fed-batch fermentation products of CGK4 in LBRCmedium at 132 h; D: The standard of 2,3-butanediol ((2S,3S)-2,3-butanediol:(2R,3R)-2,3-butanediol: meso-2,3-butanediol= 0: 98: 2). E: Fermentationproducts of CGK4 in CGXIIP medium. 1: (2S,3S)-2,3-butanediol, 2:(2R,3R)-2,3-butanediol, 3: meso-2,3-butanediol. Table S1. Primers used in this study. [file 12934_2022_1875_MOESM1_ESM.docx]

**Metabolic engineering of *Corynebacterium glutamicum* for efficient production of optically pure (2R,3R)-2,3-butanediol**

Mengyun Kou^1^, Jing Fu^2^, Wei Dai^1^, Zhenzhen Cui^1^, Zhiwen Wang^1^ and Tao Chen^1, *^

^1^ Frontier Science Center for Synthetic Biology and Key Laboratory of Systems Bioengineering of Ministry of Education, SynBio Research Platform, Collaborative Innovation Center of Chemical Science and Engineering, School of Chemical Engineering and Technology, Tianjin University, Tianjin 300072, China.

^2^ Chalmers University of Technology - Department of Biology and Biological Engineering, Gothenburg, Sweden.

* Corresponding author: Tao Chen

E-mail: chentao@tju.edu.cn

Address: Department of Biochemical Engineering, School of Chemical Engineering and Technology, Tianjin University, Tianjin 300072, People’s Republic of China.

**Additional Figures**

Figure S1 Identification of 2,3-butanediol enantiomers by GC-FID. A: The optically pure standards of (2S,3S)-2,3-butanediol, (2R,3R)-2,3-butanediol and meso-2,3-butanediol had retention times of 22.957, 23.098 and 23.973 min; B: Fermentation products of CGK1 in CGXIIP medium; C: Fed-batch fermentation products of CGK4 in LBRC medium at 132 h; D: The standard of 2,3-butanediol ((2S,3S)-2,3-butanediol: (2R,3R)-2,3-butanediol: meso-2,3-butanediol= 0: 98: 2). E: Fermentation products of CGK4 in CGXIIP medium. 1: (2S,3S)-2,3-butanediol, 2: (2R,3R)-2,3-butanediol, 3: meso-2,3-butanediol.**
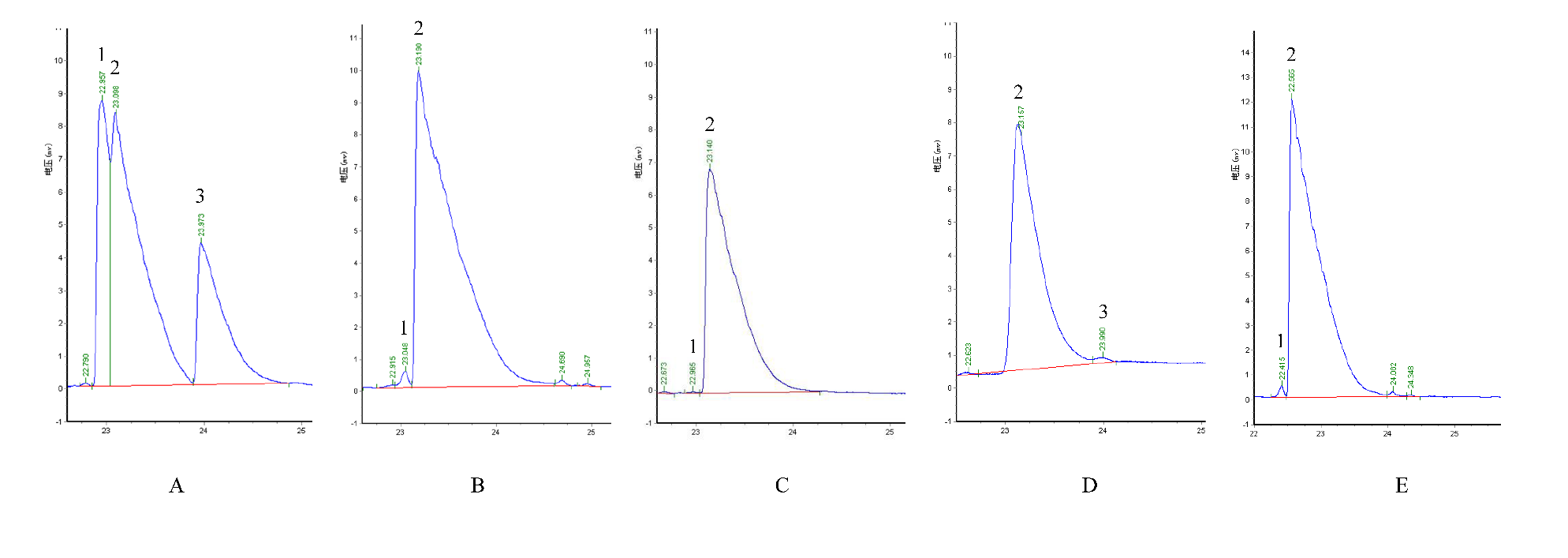
**

**Additional Tables**

**Table S1** Primers used in this study

| Primers | Sequence(5’-3’) |
| --- | --- |
| *trc*-F | GTATCCCTGCAGGCGACTGCACGGTGCACCAATGCTTC |
| *trc*-R | ATGGTTGTCCTCCTTTGAGCTCGAATTCCATGGTCTGTTTC |
| *bdhA*1-F | GAATTCGAGCTCAAAGGAGGACAACCATGAAGGCAGCAAGATGGCATAAC |
| *bdhA*1-R | GCATTGAGTCGACTTAGTTAGGTCTAACAAGGATTTTGAC |
| *Sod1*-F | GTACTCCTGCAGGTAGCTGCCAATTATTCCGGGCTTGTG |
| *Sod1*-R | CCTTTCTCGGGTTCGAATTTTTTTCTCGTGGGTAAAAAATCCTTTCGTAG |
| *bdhA*2-F | TTCGAACCCGAGAAAGGAGGTATTATGAAGGCAGCAAGATGGCATAACCAAAAGG |
| *bdhA*2-R | GCATTGAGTCGACTTAGTTAGGTCTAACAAGGATTTTG |
| *sod2*-F | GTACTCCTGCAGGTAGCTGCCAATTATTCCGGGCTTGTGA |
| *sod2*-R | CCTTTCTCGGGTTCGAATTTTTTTCTCGTGGGTAAAAAATCCTTTCGTAG |
| *bdhA*3-F | TTCGAACCCGAGAAAGGAGGTATTATGAAGGCAGCAAGATGGCATAACCAAAAGG |
| *bdhA*3-R | CTTTCTCGGGTTCGAATTTTTTTCTCGTTTAGTTAGGTCTAACAAGGATTTTGACTTGG |
| *udhA*1-F | CGAGAAAAAAATTCGAACCCGAGAAAGGAGGTATTATGCCACATTCCTACGATTACGAT |
| *udhA*1-R | GCATTGAGTCGACTTAAAACAGGCGGTTTAAACCGTTTAACG |
| *atpG*U-F | GATCCTCTAGAAATTGCGTGACCGAATTCGTT |
| *atpG*U-R | TTCATCGCGTTTCGACGTAGAGCGGACTCTGCAGCTG |
| *atpG*D-F | GCTGCAGAGTCCGCTCTACGTCGAAACGCGATGAAGTCTG |
| *atpG*D-R | CTATCAGCATGCTTCGAGGAAGAGGTCGGTGCCCTCAC |
| AU1-F | GTCAATTCAGGGTGGTGAATTAGCTGCCAATTATTCCGGGCTTGTGA |
| AU1-R | CAGGTAGACTCTAGATTAAAACAGGCGGTTTAAACCGTTTAAC |
| pEC-F | TTTAAACCGCCTGTTTTAATCTAGAGTCGACCTGCAGGCATGC |
| pEC-R | GCCCGGAATAATTGGCAGCTAATTCACCACCCTGAATTGAC |
